# Identification and Application of the Heptad Repeat Domain in the CPR5 Protein for Enhancing Plant Immunity

**DOI:** 10.1111/mpp.70059

**Published:** 2025-02-05

**Authors:** Yuehui Zhang, Yuting Ge, Keke Sun, Leiwen Pan, Zhilin Liang, Ping Wang, Yingfan Cai, Shui Wang

**Affiliations:** ^1^ Shanghai Collaborative Innovation Centre of Plant Germplasm Resources, College of Life Sciences Shanghai Normal University Shanghai China; ^2^ National Key Laboratory of Cotton Biological Breeding and Utilization, School of Life Sciences Henan University Kaifeng China; ^3^ Shanghai Songjiang Jiuting No. 3 Primary School Shanghai China; ^4^ No. 107 High School of Zhengzhou Zhengzhou China

**Keywords:** cotton Verticillium wilt, CPR5, heptad repeat domain, plant immunity

## Abstract

Plant resistance to pathogens can be significantly enhanced through genetic modification, thereby reducing the reliance on chemical pesticides. CONSTITUTIVE EXPRESSER OF PATHOGENESIS‐RELATED GENES 5 (CPR5) serves as a key negative regulator of plant immunity. Here we explored the functional domains of the CPR5 protein with the goal of dampening its activity to bolster plant immunity. Using hexapeptide asparagine–alanine–alanine–isoleucine–arginine–serine (NAAIRS) linker‐scanning analysis, we identified a heptad repeat domain (HRD) in the middle region of the CPR5 protein, which is highly conserved across the plant kingdom. The HRD is predicted to form an α‐helix structure and acts as an interface for CPR5 dimerization. Intriguingly, overexpression of the HRD in *Arabidopsis* wild‐type plants resulted in a phenotype similar to the *cpr5* mutant and led to an enhancement of plant immunity, indicating that the introduced HRDs disrupt the native CPR5 dimers, thereby relieving the suppression of plant immunity. Furthermore, expression of the HRD under the control of a pathogen‐inducible promoter significantly improved the resistance of cotton plants to *Verticillium dahliae*, a destructive wilt pathogen affecting cotton production worldwide. These findings suggest that downregulating CPR5 activity by the pathogen‐inducible expression of its HRD could be a promising approach for strengthening plant immunity.

## Graphical abstract

Plant immunity can be boosted by introducing a heptad repeat domain (HRD) of the nucleoporin CPR5 protein, which serves as an interface for CPR5 dimerization.

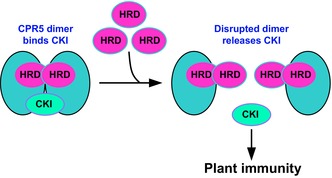

1

Plant diseases annually account for about 10% loss of global food production (Strange and Scott 2005). When plants are infected by pathogens, they mount a two‐tiered immune response: pathogen‐associated molecular pattern (PAMP)‐triggered immunity (PTI) and effector‐triggered immunity (ETI). Plant cell surface pattern recognition receptors (PRRs) detect pathogen PAMPs to initiate PTI, which serves as a foundational, nonspecific immune response effective against a wide array of pathogens. Intracellular nucleotide‐binding leucine‐rich repeat receptors (NLRs) in plants recognise pathogen effectors to trigger ETI, which is often associated with programmed cell death (PCD) and is characterised by its high specificity, typically targeting specific pathogens or strains (Jones and Dangl 2006). CONSTITUTIVE EXPRESSER OF PATHOGENESIS‐RELATED GENES 5 (CPR5) serves as a key negative regulator of plant ETI. The *cpr5* mutant was identified through a genetic screen for *constitutive expression of pathogenesis‐related genes* (*cpr*), employing the *PATHOGENESIS‐RELATED PROTEIN* 2 (*PR2*)*‐GUS* reporter. In this reporter, the *GUS* gene, which encodes β‐glucuronidase, is driven by the *PR2* promoter. The mutation causes the substitution of an amino acid residue from glycine to aspartic acid at position 420 of the CPR5 protein (CPR5^G420D^), which does not alter the subcellular localization and integrity of the CPR5 protein (Bowling et al. 1997; Gu et al. 2016; Wang et al. 2014). The G420 of the CPR5 protein is highly conserved across the plant kingdom (Figure S1a). The *cpr5* mutant exhibits characteristics of a typical lesion mimic mutant (LSM), manifesting a pleiotropic phenotype that includes dwarfism, early senescence, heightened immunity and branchless trichomes (Bowling et al. 1997; Clarke et al. 2000; Peng et al. 2020; Wang 2017). Therefore, the *cpr5* mutant could be used as a powerful genetic tool, particularly leveraging its morphological phenotype, to elucidate the signalling pathway of plant immunity, particularly plant ETI.

CPR5 protein is localised within both the nuclear envelope and nuclear speckles (Gu et al. 2016; Peng et al. 2022; Wang et al. 2014). Through comprehensive genetic analysis, we have significantly elucidated the immune signalling pathway downstream of CPR5. On one hand, CPR5 is a plant‐specific nucleoporin, which is a key component of the nuclear pore complex (NPC). In the absence of pathogen infection, CPR5 protein exists as dimers and inhibits the core cell cycle regulators cyclin‐dependent kinase inhibitors (CKIs), as well as the nucleocytoplasmic transport of immune signals. Upon pathogen infection, the activation of plant NLRs by pathogen effectors initiates a conformational change in CPR5, transitioning it from dimers to monomers. This change releases CKIs and facilitates the nucleocytoplasmic transport of immune signals, thereby triggering ETI in plants (Gu et al. 2016; Wang et al. 2014). On the other hand, CPR5 is a constituent of the RNA processing complex found within nuclear speckles. This complex encompasses the RNA splicing activator NINETEEN COMPLEX (NTC) and the RNA polyadenylation factor CLEAVAGE AND POLYADENYLATION SPECIFICITY FACTOR (CPSF). Nuclear speckles serve as a central hub for RNA processing activities. The CPR5‐NTC/CPSF signalling pathway plays a role in alternative RNA splicing and polyadenylation, which are processes capable of increasing the diversity of plant immune signals as a response to ever‐evolving variety of pathogens (Peng et al. 2022). These findings suggest that the CPR5 signalling cascade acts as a central nexus, linking essential biological processes, including nuclear transport, cell cycle progression and RNA processing to promptly and efficiently modulate plant immunity.

The CPR5 protein is predicted to contain five transmembrane (TM) domains within its carboxyl (C)‐terminal region (Wang et al. 2014). Additionally, it has been identified as a member of the Transformer 2 (Tra2) subfamily of the serine/arginine‐rich (SR) family, characterised by the presence of an SR domain and an RNA recognition motif (RRM) domain in its amino (N)‐terminal region (Peng et al. 2022). Beyond these known domains, no other functionally characterised domains were identified within the CPR5 protein, particularly in its central region. To probe the essential domain or amino acid residues that are crucial for CPR5 function within its uncharacterized region, we employed the hexapeptide asparagine–alanine–alanine–isoleucine–arginine–serine (NAAIRS) linker to scan the N‐terminal region of CPR5 protein (1–340 amino acids). This hexapeptide serves as a flexible linker, adopting both α‐helical and β‐sheet conformations and is utilised for mutagenesis studies by replacing six amino acids at a time. This approach is based on the prediction that substituting with the NAAIRS sequence would be compatible with the native protein's three‐dimensional structure (Wilson et al. 1985).

The *CPR5* gene, approximately 4.25 kb in length, used in this study is biologically functional, capable of fully complementing the *cpr5* mutant phenotype (Figure 1a and Figure S1b,c). CPR5 proteins are highly conserved throughout the plant kingdom (Figures S1a and S2). The CPR5 NAAIRS analysis was abbreviated as CNA. Among the conserved regions in the N‐terminus of CPR5 protein, we generated 40 CNA constructs (CNA1–CNA40), each incorporating the NAAIRS‐encoding DNA sequence (5′‐AATGCTGCTATACGATCG‐3′) into the corresponding region of the *CPR5* gene (for methods used, see File S1). These constructs were subsequently introduced into the *cpr5* mutant to evaluate the effect of the hexapeptide substitution on CPR5 function (Figure 1b,c; Figures S2 and S3).

**FIGURE 1:**
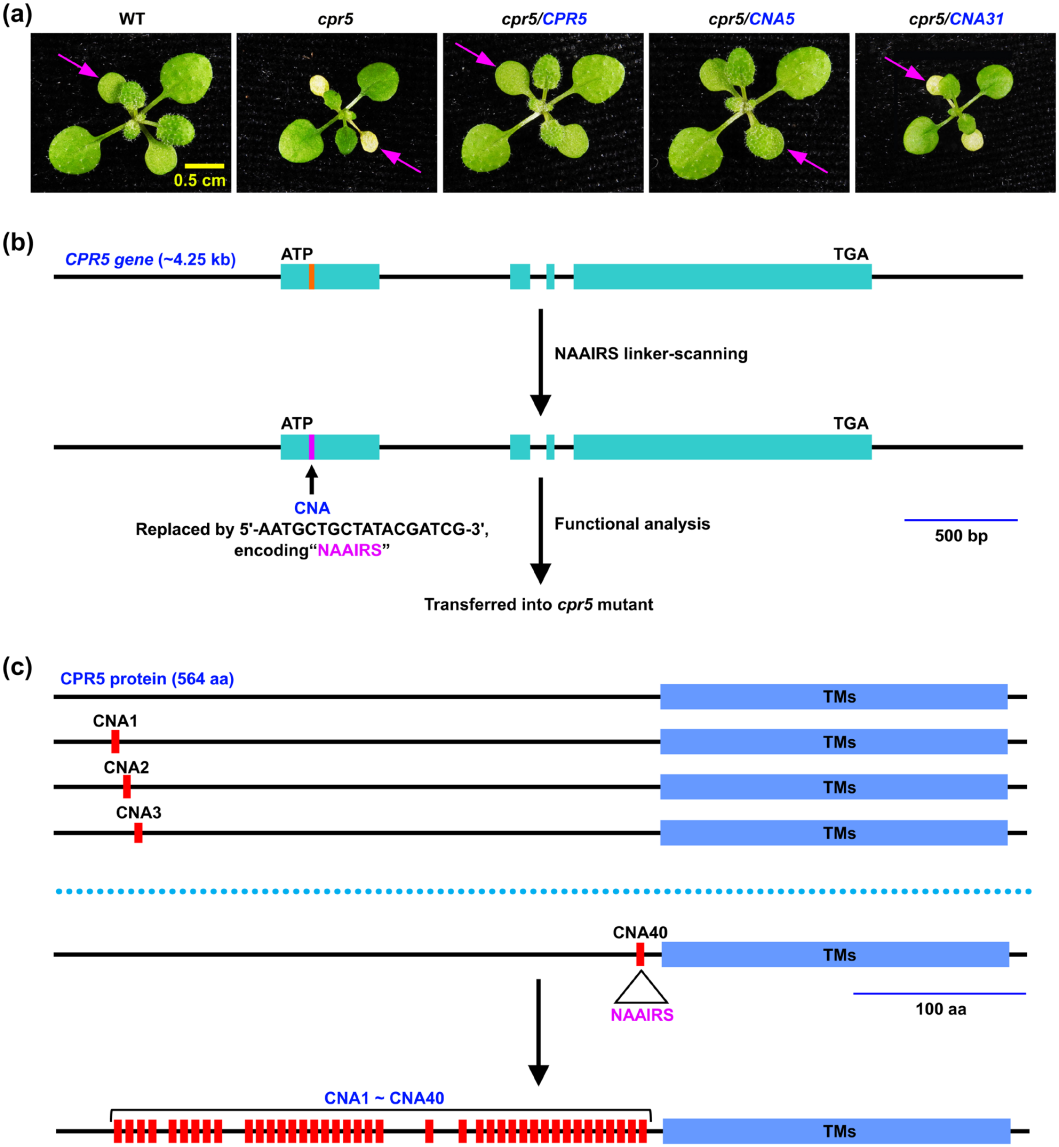
CPR5 NAAIRS analysis (CNA). (a) Two‐week‐old wild‐type (WT), *cpr5*, *cpr5*/*CPR5* (*cpr5* transformed with the *CPR5* gene), *cpr5*/*CNA5* (*cpr5* transformed with the *CNA5* construct) and *cpr5*/*CNA31* (*cpr5* transformed with the *CNA31* construct) plants were photographed for early senescence (arrows). The *CPR5* gene spans approximately 4.25 kilobases (kb) and includes a 1‐kb promoter region (located upstream of the initiation codon ATG), exons, introns and a 668‐base pair (bp) terminal region (located downstream of the termination codon TGA). (b) The CNA constructs were generated by replacing an 18‐bp nucleotide sequence in the *CPR5* gene with the sequence 5′‐AATGCTGCTATACGATCG‐3′, which encodes the hexapeptide asparagine–alanine–alanine–isoleucine–arginine–serine (NAAIRS) and serves a linker to scan the essence of amino acid residues in the CPR5 protein. These constructs were subsequently introduced into *cpr5* mutants to evaluate the critical role of the six substituted amino acids in the function of CPR5. (c) A total of 40 CNA constructs (CNA1–CNA40) were produced, spanning the N‐terminal conserved region of the CPR5 protein. TMs, transmembrane domains.

Based on their ability to rescue the *cpr5* mutant phenotype, hexapeptide regions substituted with NAAIRS were classified into two groups: those considered essential for the CPR5 protein, which failed to complement the *cpr5* mutant, and those regarded as non‐essential, which successfully complemented the mutant. Of the 40 CNA constructs, the regions encompassing eight constructs, specifically CNA31 to CNA35 and CNA37 to CNA39, were identified as essential, whereas the rest were classified as nonessential. Detailed analysis uncovered a heptad repeat domain (HRD), which is highly conserved in plants, embedded within the essential regions (Figures 1a and 2a,b; Table 1). This HRD adopts an amphipathic α‐helix structure. The α‐helix serves as a primary motif for protein subunit oligomerization, characterised by a heptad repeat pattern predominantly composed of apolar residues that form the oligomer interface (Burkhard, Stetefeld, and Strelkov 2001). The crucial role of these apolar residues was further validated by point mutation analysis, which demonstrated that mutations at positions L284, L291 and L305 resulted in the loss of CPR5 function (Table 1). In agreement with this, a high‐confidence prediction of a coiled coil (CC) domain within this region (amino acids 267–341) is presented (Figure 2c and Figure S4a).

**FIGURE 2:**
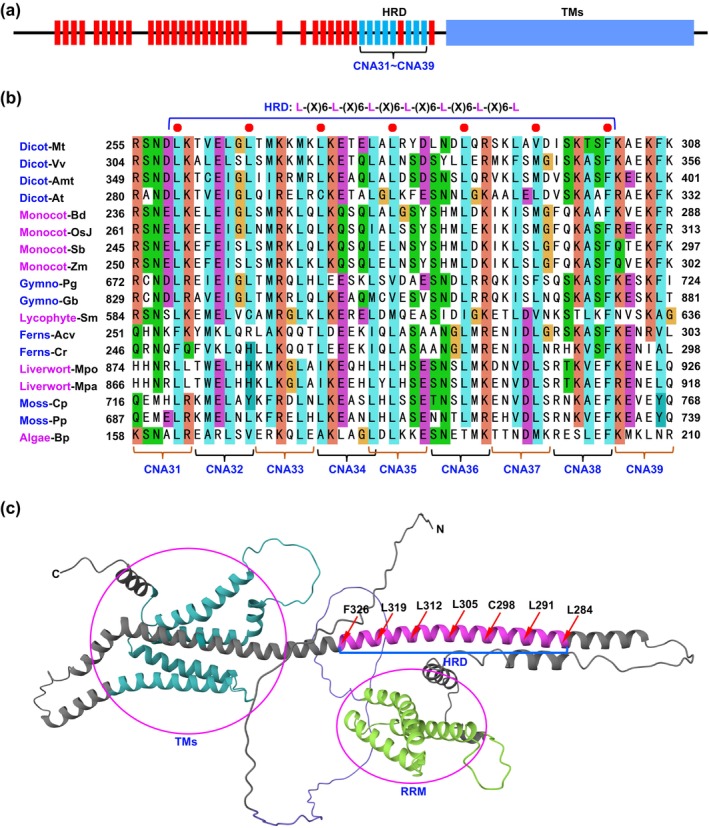
Heptad repeat domain (HRD) is essential for CPR5 protein. (a) The schematic diagram illustrates the result of the CPR5 NAAIRS analysis. The red bars indicate the substitution of NAAIRS did not impact the CPR5 function, while the blue bars indicate that the substitution of NAAIRS disrupted the CPR5 function. TMs, transmembrane domains. (b) Alignments of plant HRDs, including algae *Bathycoccus prasinos* (*Bp*); mosses *Ceratodon purpureus* (*Cp*) and *Physcomitrium patens* (*Pp*); liverworts *Marchantia paleacea* (*Mpa*) and *Marchantia polymorpha* (*Mpo*); ferns *Adiantum capillus‐veneris* (*Acv*) and *Ceratopteris richardii* (*Cr*); lycophyte *Selaginella moellendorffii* (*Sm*); gymnosperms *Picea glauca* (*Pa*) and *Ginkgo biloba* (*Gb*); monocots *Brachypodium distachyon* (*Bd*), *Oryza sativa Japonica* (*OsJ*), *Sorghum bicolor* (*Sb*) and *Zea mays* (*Zm*); dicots *Amborella trichopoda* (*Amt*), *Arabidopsis thaliana* (*At*), *Medicago truncatula* (*Mt*) and *Vitis vinifera* (*Vv*). The red dots indicate the conserved apolar amino acid residues in the HRDs (L‐(X)_6_‐L‐(X)_6_‐L‐(X)_6_‐L‐(X)_6_‐L‐(X)_6_‐L‐(X)_6_‐L). The positions of CNA31–CNA39 are indicated. (c) The three‐dimensional structure of CPR5 protein, as predicted by AlphaFold, is available at https://www.alphafold.ebi.ac.uk/. The arrows denote the conserved apolar amino acid residues in the HRDs. RRM, RNA recognition motif; TMs, transmembrane domains.

**TABLE 1:** Results of the CPR5 NAAIRS analysis and the point mutation.

Construct	Type of replacement	Position	Transgenic plants			Essence to CPR5 function
			Background	Number of T1 plants	Phenotype	
CNA1	NAAIRS	38–43 aa	*cpr5*	25	WT	No
CNA2	NAAIRS	44–49 aa	*cpr5*	21	WT	No
CNA3	NAAIRS	50–55 aa	*cpr5*	22	WT	No
CNA4	NAAIRS	56–61 aa	*cpr5*	24	WT	No
CNA5	NAAIRS	68–73 aa	cpr5	32	WT	No
CNA6	NAAIRS	74–79 aa	*cpr5*	38	WT	No
CNA7	NAAIRS	80–85 aa	*cpr5*	27	WT	No
CNA8	NAAIRS	86–91 aa	*cpr5*	27	WT	No
CNA9	NAAIRS	92–97 aa	*cpr5*	25	WT	No
CNA10	NAAIRS	116–121 aa	*cpr5*	26	WT	No
CNA11	NAAIRS	122–127 aa	*cpr5*	22	WT	No
CNA12	NAAIRS	128–133 aa	*cpr5*	24	WT	No
CNA13	NAAIRS	134–139 aa	*cpr5*	21	WT	No
CNA14	NAAIRS	140–145 aa	*cpr5*	22	WT	No
CNA15	NAAIRS	146–151 aa	*cpr5*	26	WT	No
CNA16	NAAIRS	151–156 aa	*cpr5*	22	WT	No
CNA17	NAAIRS	157–162 aa	*cpr5*	26	WT	No
CNA18	NAAIRS	162–167 aa	*cpr5*	29	WT	No
CNA19	NAAIRS	168–173 aa	*cpr5*	30	WT	No
CNA20	NAAIRS	174–179 aa	*cpr5*	28	WT	No
CNA21	NAAIRS	179–184 aa	*cpr5*	35	WT	No
CNA22	NAAIRS	185–190 aa	*cpr5*	33	WT	No
CNA23	NAAIRS	215–220 aa	*cpr5*	28	WT	No
CNA24	NAAIRS	233–238 aa	*cpr5*	20	WT	No
CNA25	NAAIRS	244–249 aa	*cpr5*	21	WT	No
CNA26	NAAIRS	250–255 aa	*cpr5*	32	WT	No
CNA27	NAAIRS	256–261 aa	*cpr5*	24	WT	No
CNA28	NAAIRS	262–267 aa	*cpr5*	27	WT	No
CNA29	NAAIRS	268–273 aa	*cpr5*	25	WT	No
CNA30	NAAIRS	274–279 aa	*cpr5*	25	WT	No
CNA31	NAAIRS	280–285 aa	*cpr5*	37	*cpr5*	Yes
CNA32	NAAIRS	286–291 aa	*cpr5*	38	*cpr5*	Yes
CNA33	NAAIRS	292–297 aa	*cpr5*	32	*cpr5*	Yes
CNA34	NAAIRS	298–303 aa	*cpr5*	29	*cpr5*	Yes
CNA35	NAAIRS	303–308 aa	*cpr5*	23	*cpr5*	Yes
CNA36	NAAIRS	309–314 aa	*cpr5*	30	WT	No
CNA37	NAAIRS	315–320 aa	*cpr5*	21	*cpr5*	Yes
CNA38	NAAIRS	321–326 aa	*cpr5*	33	*cpr5*	Yes
CNA39	NAAIRS	327–332 aa	*cpr5*	35	*cpr5*	Yes
CNA40	NAAIRS	333–338 aa	*cpr5*	24	WT	No
HRD‐M‐1	Point mutation	L284R	*cpr5*	25	*cpr5*	Yes
HRD‐M‐2	Point mutation	L291R	*cpr5*	28	*cpr5*	Yes
HRD‐M‐3	Point mutation	C298R	*cpr5*	25	WT	No
HRD‐M‐4	Point mutation	L305R	*cpr5*	27	*cpr5*	Yes
HRD‐M‐5	Point mutation	L312R	*cpr5*	32	WT	No
HRD‐M‐6	Point mutation	L319R	*cpr5*	35	WT	No
HRD‐M‐7	Point mutation	L326R	*cpr5*	28	WT	No

Abbreviations: aa, amino acid; CNA, CPR5 NAAIRS analysis; HRD‐M, point mutation of the apolar residue in the heptad repeat domain (HRD) from leucine (L)/cysteine (C) to arginine (R); NAAIRS, hexapeptide asparagine–alanine–alanine–isoleucine–arginine–serine.

To detect the subcellular localization of the HRD of CPR5 protein (CPR5‐HRD), we generated the fluorescent fusion protein of VENUS and CPR5‐HRD (VENUS‐CPR5‐HRD) and demonstrated that CPR5‐HRD was localised in both the cytoplasm and the nucleus (Figure 3a). It has been previously shown that, in the absence of pathogen infection, CPR5 proteins form dimers (Gu et al. 2016). As shown in Figure 3b, the co‐immunoprecipitation (CoIP) analysis demonstrated that the CPR5 dimers were significantly disrupted by the introduction of its HRD. We subsequently overexpressed the HRD region of the *CPR5* gene in wild‐type (WT) plants (Figure S4b). Interestingly, the overexpression lines displayed a *cpr5* mutant phenotype, and accordingly, the defence marker genes *PR1* and *PR2* were significantly upregulated to levels comparable with those in *cpr5* mutants (Figure 3c,d). Analysis of pathogen infection revealed that the HRD‐overexpressing lines exhibited resistance akin to that of the *cpr5* mutants against both the virulent pathogen *Pseudomonas syringae* pv. *maculicola* ES4326 (Psm) and the avirulent pathogen Psm/*AvrRpt2* (Psm harbouring the effector gene *AvrRpt2*) (Figure 3e). These findings suggest that the HRD may serve as an interface for CPR5 oligomerization and that the HRD alone (when truncated to include only this domain) is capable of binding to and disrupting the dimer formation and function of endogenous CPR5 proteins, thereby resulting in the *cpr5* mutant phenotype.

**FIGURE 3:**
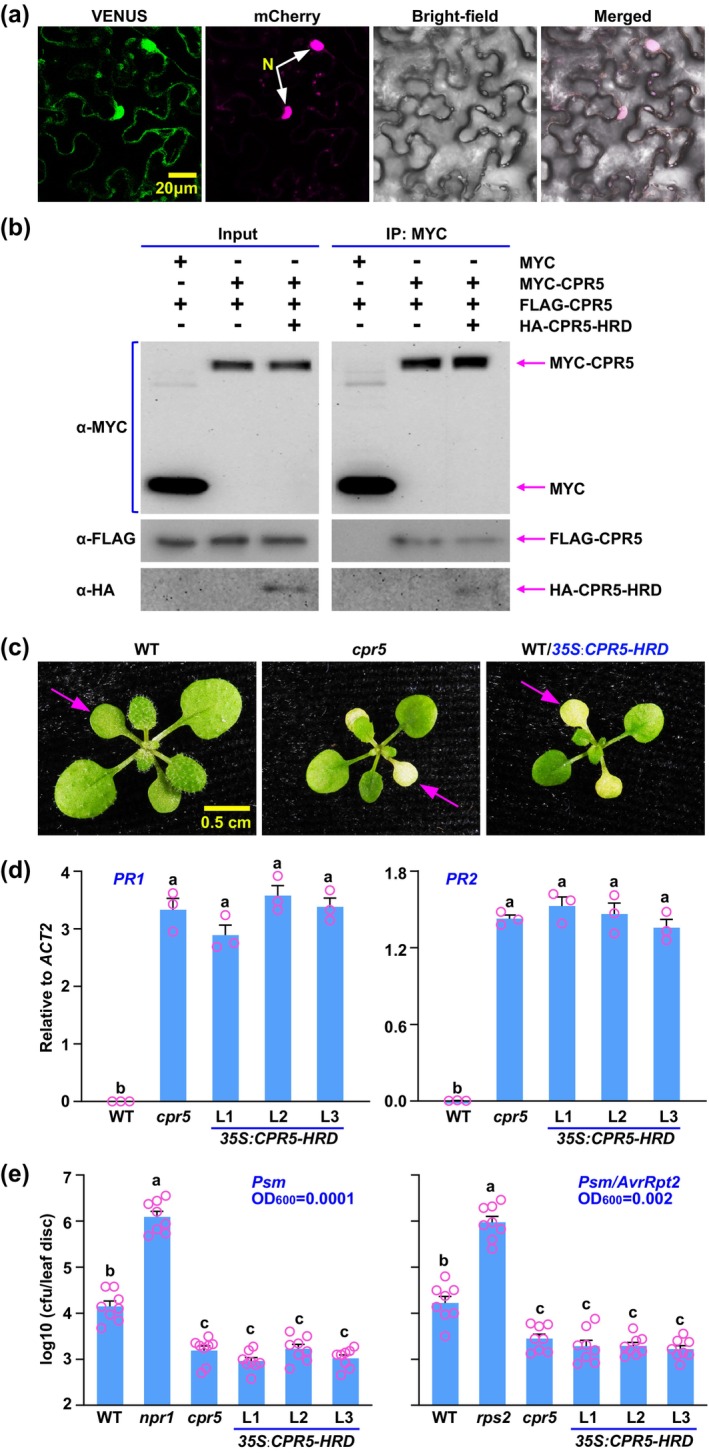
Heptad repeat domain (HRD) of the CPR5 protein enhances resistance to pathogen infection in *Arabidopsis*. (a) Subcellular localization was conducted by transiently coexpressing the VENUS‐tagged HRD of CPR5 protein (*35S‐VENUS‐CPR5‐HRD*) with mCherry‐NLS (where the nuclear localization signal of SV40 protein is fused to the C‐terminus of mCherry) in *Nicotiana benthamiana* for 2 days. The mCherry‐NLS served as a nuclear marker. N, nucleus. (b) Co‐immunoprecipitation was carried out by transiently co‐expressing MYC‐tagged CPR5 (MYC‐CPR5) and FLAG‐tagged CPR5 (FLAG‐CPR5) along with or without hemagglutinin (HA)‐tagged HRD of CPR5 protein (HA‐CPR5‐HRD) in *N. benthamiana* for 2 days. Protein extracts were immunoprecipitated (IPed) using anti‐MYC (α‐MYC) antibody and resolved by SDS‐PAGE. Both input and IPed proteins were detected using α‐MYC, anti‐FLAG (α‐FLAG) and anti‐HA (α‐HA) antibodies. (c) Two‐week‐old wild‐type (WT), *cpr5* and WT/*35S: CPR5‐HRD* (WT transformed with *35S: CPR5‐HRD*) *Arabidopsis* plants were photographed for early senescence (arrows). (d) Reverse transcription‐quantitative PCR was carried out on *PR1* (Left panel) and *PR2* (Right panel) in 2‐week‐old WT, *cpr5* and WT/*35S: CPR5‐HRD* (three lines: L1, L2 and L3) *Arabidopsis* plants. *ACT*2 was used as an internal control. The experiments were conducted in triplicate. Data are represented as mean ± SEM (*n* = 3). Statistical differences are indicated with letters (*p* < 0.01, one‐way analysis of variance (ANOVA) with Bonferroni post hoc test). (e) Four‐week‐old WT, *cpr5* and WT/*35S: CPR5‐HRD* (three lines: L1, L2, and L3) *Arabidopsis* plants were inoculated with the virulent pathogen *Pseudomonas syringae* pv. *maculicola* ES4326 (Psm, OD_600_ = 0.0001) (left panel) or the avirulent pathogen Psm/*AvrRpt2* (‐Psm carrying the effector gene *AvrRpt2* , OD_600_ = 0.002) (right panel). Mutants of *npr1* and *rps2* served as negative controls for Psm and Psm/*AvrRpt2*, respectively. Bacterial growth (colony‐forming units, cfu) was measured 3 days post‐inoculation (dpi). Experiments were conducted three times with similar results. Data are represented as mean ± SEM (*n* = 8). Statistical differences are indicated with letters (*p* < 0.01, one‐way ANOVA with Bonferroni post hoc test).

Based on these findings, we can harness the HRD to enhance the immunity of plants, especially crop species, because CPR5 acts as a negative regulator of plant immunity (Bowling et al. 1997; Gu et al. 2016; Wang et al. 2014). We generated a *PR1: CPR5‐HRD* construct, in which the HRD region of the *Arabidopsis CPR5* gene is driven by the *Arabidopsis PR1* promoter, a pathogen‐inducible promoter (Lebel et al. 1998). This construct was subsequently introduced into upland cotton *Gossypium hirsutum* accession HM‐1. It is widely recognised that Verticillium wilt, caused by the soilborne fungus *Verticillium dahliae*, ranks among the most devastating diseases affecting cotton production globally (Zhu et al. 2023). Our analysis of pathogen infection demonstrated that the *PR1: CPR5‐HRD*‐transgenic lines exhibited significantly greater resistance to *V. dahliae* than the WT plants (Figure 4a–c). In contrast, the plant development, including the plant height and the fruit branch angle, of the *PR1: CPR5‐HRD*‐transgenic lines was not notably affected (Figure S5a,b).

**FIGURE 4:**
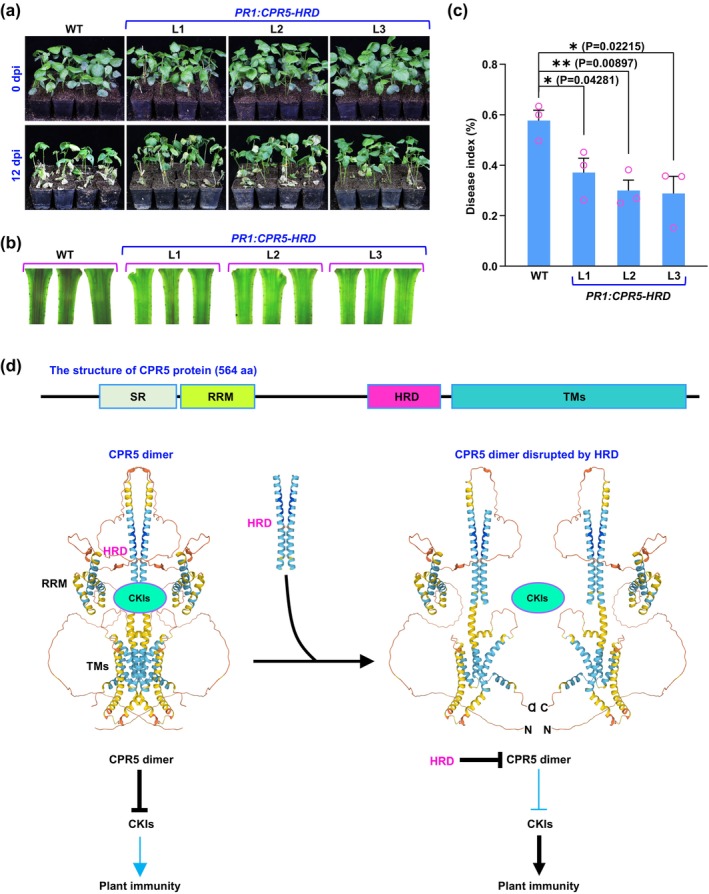
Heptad repeat domain (HRD) of the CPR5 protein enhances resistance to pathogen infection in cotton. (a) Four‐week‐old wild‐type (WT) upland cotton *Gossypium hirsutum* accession HM‐1 (used as control) and *PR1: CPR5‐HRD* (three lines: L1, L2 and L3) cotton plants with one pair of true leaves were inoculated with *Verticillium dahliae* (at a concentration of 10^7^ spores/mL) and were photographed on the inoculation day (top) and 12 days post‐inoculation (dpi) (bottom) to observe the wilt phenotype. Experiments were conducted three times with similar results. (b) Vascular tissues of stem sections from 4‐week‐old WT HM‐1 (used as control) and *PR1: CPR5‐HRD* (three lines: L1, L2 and L3) cotton plants with one pair of true leaves inoculated with *V. dahliae* (at a concentration of 10^7^ spores/mL) were photographed using a stereomicroscope at 12 dpi. Experiments were conducted three times with similar results. (c) The disease index of 4‐week‐old WT HM‐1 (used as control) and *PR1: CPR5‐HRD* (three lines: L1, L2 and L3) cotton plants with one pair of true leaves inoculated with *V. dahliae* (at a concentration of 10^7^ spores/mL) was assessed at 12 dpi. The experiments were conducted in triplicate. Data are represented as mean ± SEM (*n* = 3). Data were analysed using the two‐tailed Student's *t*‐test. NS, not significant; *p < 0.05, **p < 0.01, ***p < 0.001, ****p < 0.0001. (d) Top panel: A schematic diagram illustrating the structure of the CPR5 protein. Bottom panel: A proposed model. CPR5 proteins assemble into dimers via the interface of the HRD, thereby inhibiting the cell cycle regulators CKIs and suppressing plant immunity. Introduction of exogenous HRDs can bind to and dismantle the native CPR5 dimers, thereby freeing CKIs to stimulate plant immunity. C, C‐terminus; N, N‐terminus; RRM, RNA recognition motif; SR, serine/arginine‐rich domain; TMs, transmembrane domains.

CPR5 plays a negative role in plant immunity, preventing plants from the harm caused by unnecessary or excessive immune responses (Gu et al. 2016, Wang et al. 2014). Through the NAAIRS linker‐scanning analysis, we identified a conserved HRD in the middle region of the CPR5 protein, which probably serves as a dimerization interface for CPR5 (Figures 1 and 2; Figures S1–, S4). It has been demonstrated that, in the absence of pathogen infection, CPR5 proteins form dimers to inhibit cell cycle regulators CKIs and suppress plant immunity. Upon pathogen infection, activated NLRs induce a conformational change in CPR5, transitioning it from dimers to monomers, thereby releasing CKI and alleviating suppression of plant immunity (Gu et al. 2016). As illustrated in Figure 4d, the HRD can be employed to disrupt the endogenous CPR5 dimers and restrain their activity as it serves as a binding platform for the oligomerization of CPR5 proteins. Consistently, overexpression of the HRD in *Arabidopsis* wild‐type plants resulted in a *cpr5* mutant phenotype and enhanced plant immunity (Figure 3). Building on this discovery, we have applied it to enhance cotton resistance. Intriguingly, the *PR1: CPR5‐HRD*‐transgenic cotton lines exhibited significantly improved resistance, and more importantly, no adverse effect on plant development (Figure 4a and Figure S5‐c). Thus, genetically reducing CPR5 activity by the pathogen‐inducible expression of its HRD appears to be a promising strategy for bolstering plant immunity, with potential application beyond *Arabidopsis* and cotton.

## Conflicts of Interest

The authors declare no conflicts of interest.

## Supporting information


**FIGURE S1.** The *cpr5* mutant is complemented by the *CPR5* gene. (a) Top panel: The structure of the CPR5 protein. SR, Serine/arginine‐rich domain; RRM, RNA recognition motif; TM, transmembrane domain (TM1–TM5). Bottom panel: The amino acid residue at position 420 in the cpr5 protein is altered from glycine (G) to aspartic acid (D) (CPR5^G420D^) and is highly conserved across the plant kingdom. Alignment of the TM2 domain of plant CPR5 proteins from dicots including *Arabidopsis thaliana* , *Medicago truncatula* , and *Populus trichocarpa* ; monocots including *Hordeum vulgare* , *Oryza sativa japonica*, and *Zea mays* ; gymnosperms including *Larix laricina* and *Picea glauca* ; liverworts including *Marchantia paleacea* and *Marchantia polymorpha* ; ferns including *Adiantum nelumboides* and *Ceratopteris richardii* ; lycophytes incuding *Isoetes echinospora* and *Selaginella moellendorffii*; mosses including *Physcomitrella patens* and *Sphagnum fallax* ; algae including *Klebsormidium nitens* and *Ostreococcus tauri*. (b) Top panel: 2‐week‐old wild‐type (WT), *cpr5*, and *cpr5*/*CPR5* (the *cpr5* mutant transformed with the 4.25‐kb *CPR5* gene) plants. Bottom panel: The scanning electron microscopy (SEM) images of trichomes on leaves of WT, *cpr5*, and *cpr5*/*CPR5* plants. (c) Reverse transcription‐quantitative PCR was carried out on *PR1* and *PR2* in 2‐week‐old WT, *cpr5*, and three lines of *cpr5*/*CPR5* (L1–L3) plants. *ACT2* was used as an internal control. Data are represented as mean ± SEM (*n* = 3). Statistical differences are indicated with letters (*p* < 0.01, one‐way ANOVA with Bonferroni post hoc test).

**FIGURE S2.** Alignment of dicot CPR5 proteins. Plants include *Arabidopsis thaliana* (*At*), *Gossypium arboretum* (*Ga*), *Ipomoea triloba* (*It*), *Nicotiana attenuata* (*Na*), *Populus trichocarpa* (*Pt*), *Prunus mume* (*Pm*), *Ricinus communis* (*Rc*), *Solanum pennellii* (*Sp*), *Spinacia oleracea* (*So*), *Vitis vinifera* (*Vv*). The locations of the NAAIRS substitutions are indicated. CNA, CPR5 NAAIRS analysis; TM, transmembrane domain.

**FIGURE S3.** The locations of the NAAIRS substitutions in the CPR5 protein sequence.

**FIGURE S4.** The HRD of CPR5 protein. (a) A coiled coil domain in CPR5 protein is predicted by COILS at https://embnet.vital‐it.ch/software/COILS_form.html. (b) Two‐week‐old WT, *cpr5*, and three lines of *35S‐CPR5‐HRD*‐transgenic (L1–L3) plants were used to perform western blot analysis. The CPR5‐HRD protein was tagged with hemagglutinin (HA). Total proteins were blotted with anti‐HA antibody. Anti‐α‐tubulin antibody served as a loading control.

**FIGURE S5.** The phenotype of *PR1: CPR5‐HRD*‐transgenic cotton plants. (a) The height of 60‐day‐old wild‐type (WT) and three lines of *PR1: CPR5‐HRD*‐transgenic (L1–L3) cotton plants. (b) The fruit branch angle of 60‐day‐old WT and three lines of *PR1: CPR5‐HRD*‐transgenic (L1–L3) cotton plants.

**FILE S1.** Experimental procedures.

**TABLE S1.** The primers used for this study.

## Data Availability

The data that support the findings of this study are available from the corresponding author upon reasonable request.
