# Supplementary figures and images for: Identification and Application of the Heptad Repeat Domain in the CPR5 Protein for Enhancing Plant Immunity
Source: Mol Plant Pathol. 2025 Feb 5;26(2):e70059. doi: 10.1111/mpp.70059 (PMC11798864; doi:10.1111/mpp.70059)

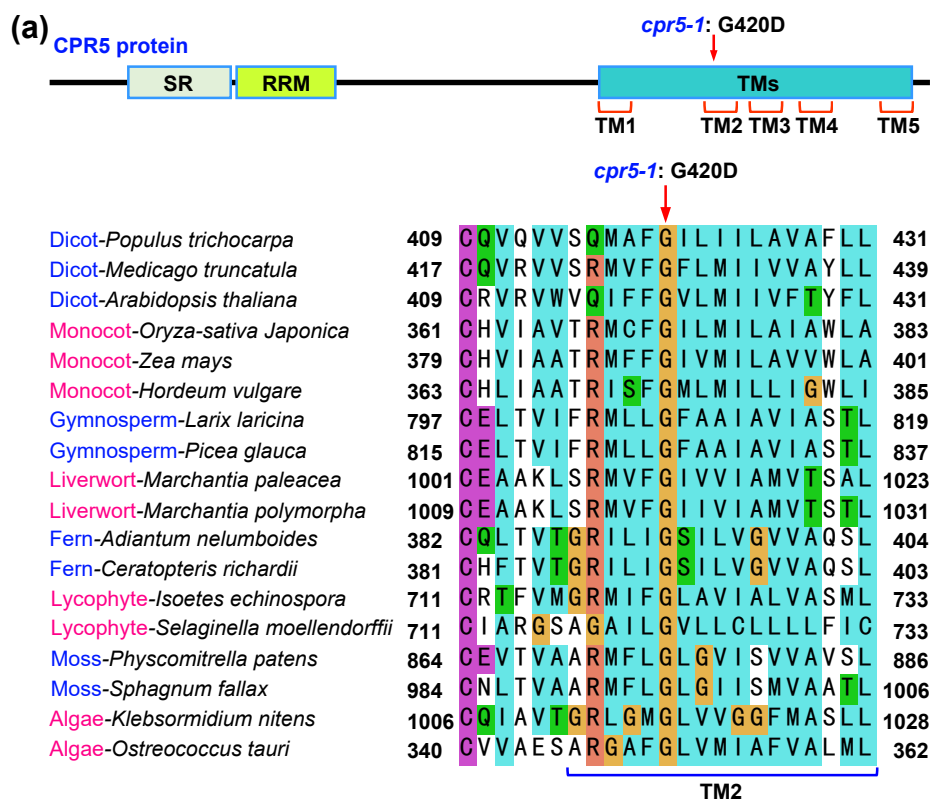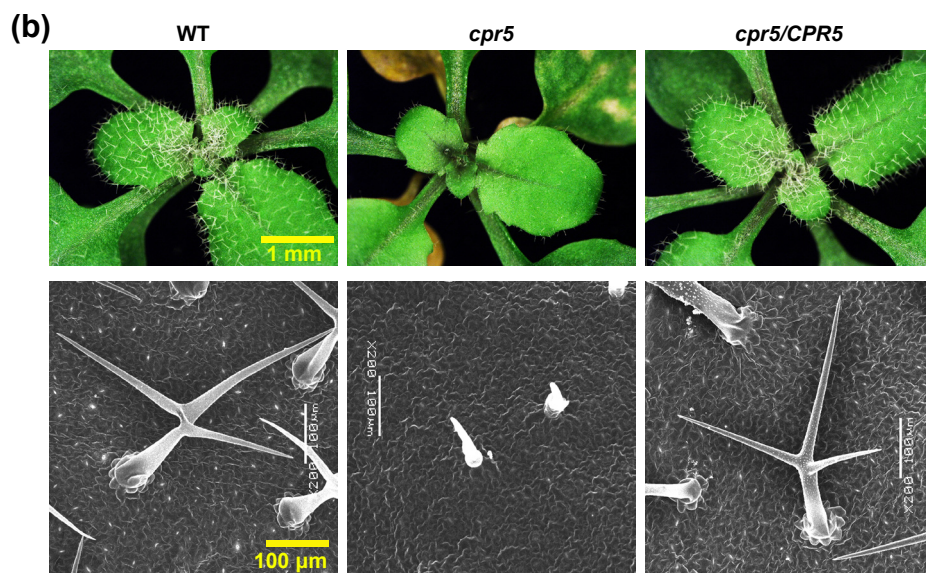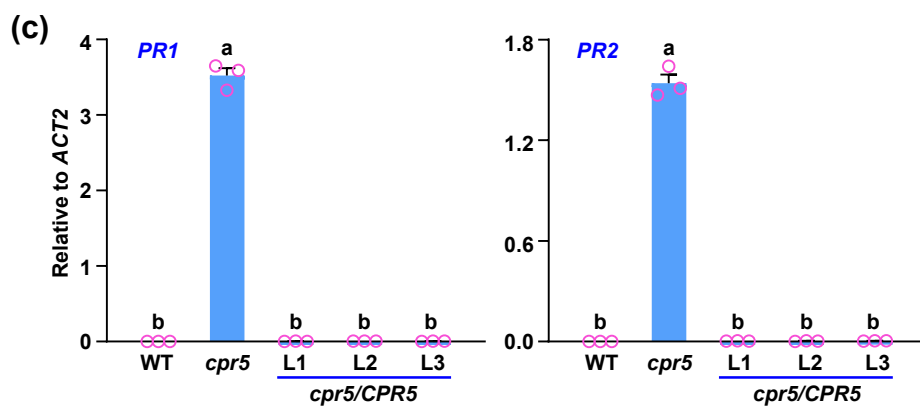

Supplement: Supplementary file 1 — FIGURE S1. The cpr5 mutant is complemented by the CPR5 gene. (a) Top panel: The structure of the CPR5 protein. SR, Serine/arginine‐rich domain; RRM, RNA recognition motif; TM, transmembrane domain (TM1–TM5). Bottom panel: The amino acid residue at position 420 in the cpr5 protein is altered from glycine (G) to aspartic acid (D) (CPR5G420D) and is highly conserved across the plant kingdom. Alignment of the TM2 domain of plant CPR5 proteins from dicots including Arabidopsis thaliana , Medicago truncatula , and Populus trichocarpa ; monocots including Hordeum vulgare , Oryza sativa japonica, and Zea mays ; gymnosperms including Larix laricina and Picea glauca ; liverworts including Marchantia paleacea and Marchantia polymorpha ; ferns including Adiantum nelumboides and Ceratopteris richardii ; lycophytes incuding Isoetes echinospora and Selaginella moellendorffii; mosses including Physcomitrella patens and Sphagnum fallax ; algae including Klebsormidium nitens and Ostreococcus tauri. (b) Top panel: 2‐week‐old wild‐type (WT), cpr5, and cpr5/CPR5 (the cpr5 mutant transformed with the 4.25‐kb CPR5 gene) plants. Bottom panel: The scanning electron microscopy (SEM) images of trichomes on leaves of WT, cpr5, and cpr5/CPR5 plants. (c) Reverse transcription‐quantitative PCR was carried out on PR1 and PR2 in 2‐week‐old WT, cpr5, and three lines of cpr5/CPR5 (L1–L3) plants. ACT2 was used as an internal control. Data are represented as mean ± SEM (n = 3). Statistical differences are indicated with letters (p < 0.01, one‐way ANOVA with Bonferroni post hoc test). [file MPP-26-e70059-s003.pdf]

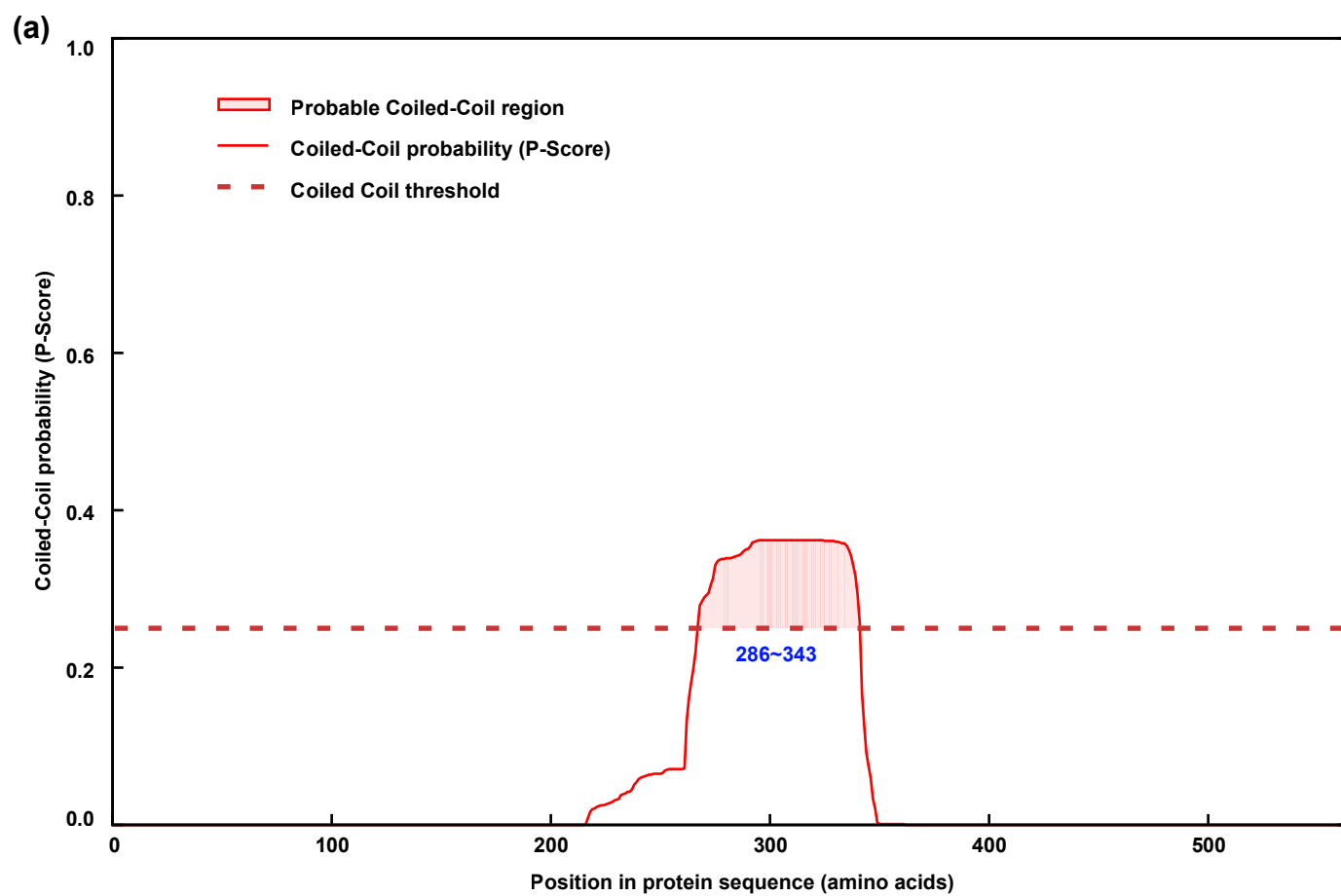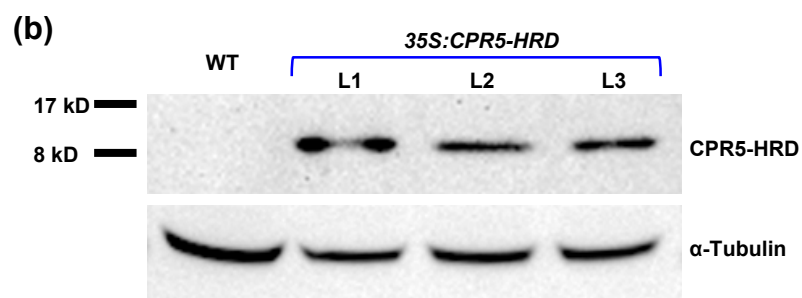

Supplement: Supplementary file 4 — FIGURE S4. The HRD of CPR5 protein. (a) A coiled coil domain in CPR5 protein is predicted by COILS at https://embnet.vital‐it.ch/software/COILS_form.html. (b) Two‐week‐old WT, cpr5, and three lines of 35S‐CPR5‐HRD‐transgenic (L1–L3) plants were used to perform western blot analysis. The CPR5‐HRD protein was tagged with hemagglutinin (HA). Total proteins were blotted with anti‐HA antibody. Anti‐α‐tubulin antibody served as a loading control. [file MPP-26-e70059-s006.pdf]

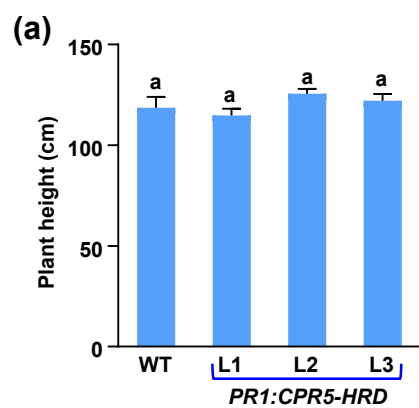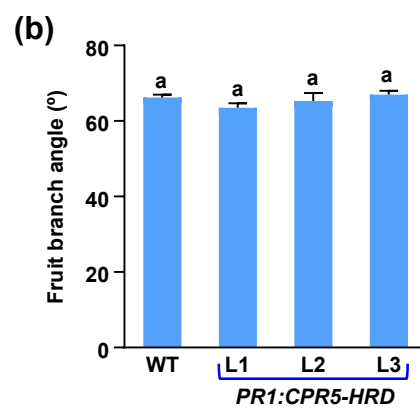

Supplement: Supplementary file 5 — FIGURE S5. The phenotype of PR1: CPR5‐HRD‐transgenic cotton plants. (a) The height of 60‐day‐old wild‐type (WT) and three lines of PR1: CPR5‐HRD‐transgenic (L1–L3) cotton plants. (b) The fruit branch angle of 60‐day‐old WT and three lines of PR1: CPR5‐HRD‐transgenic (L1–L3) cotton plants. [file MPP-26-e70059-s004.pdf]
