# Supplementary material for: Identification and Application of the Heptad Repeat Domain in the CPR5 Protein for Enhancing Plant Immunity
Source: Mol Plant Pathol. 2025 Feb 5;26(2):e70059. doi: 10.1111/mpp.70059 (PMC11798864; doi:10.1111/mpp.70059)
Supplement: Supplementary file 2 — FIGURE S2. Alignment of dicot CPR5 proteins. Plants include Arabidopsis thaliana (At), Gossypium arboretum (Ga), Ipomoea triloba (It), Nicotiana attenuata (Na), Populus trichocarpa (Pt), Prunus mume (Pm), Ricinus communis (Rc), Solanum pennellii (Sp), Spinacia oleracea (So), Vitis vinifera (Vv). The locations of the NAAIRS substitutions are indicated. CNA, CPR5 NAAIRS analysis; TM, transmembrane domain. [file MPP-26-e70059-s001.pdf]

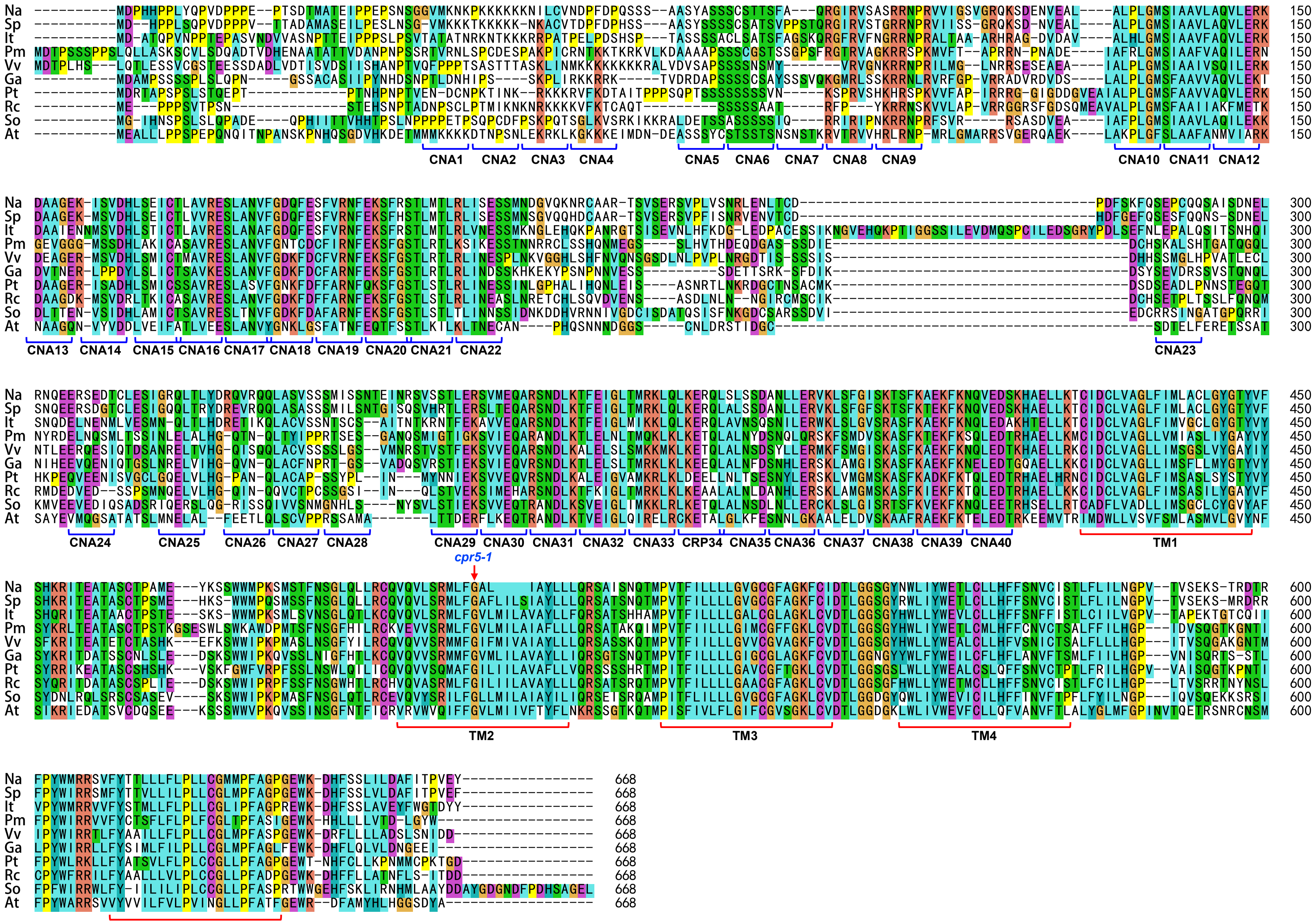

Na
Sp
It
Pm
Vv
Ga
Pt
Rc
So
At
150
CNA1 CNA2 CNA3 CNA4 CNA5 CNA6 CNA7 CNA8 CNA9 CNA10 CNA11 CNA12
300
CNA13 CNA14 CNA15 CNA16 CNA17 CNA18 CNA19 CNA20 CNA21 CNA22 CNA23
450
CNA24 CNA25 CNA26 CNA27 CNA28 CNA29 CNA30 CNA31 CNA32 CNA33 CRP34 CNA35 CNA36 CNA37 CNA38 CNA39 CNA40
TM1
cpr5-1
600
TM2
TM3
TM4
668
TM5
