# Supplementary material for: Identification and Application of the Heptad Repeat Domain in the CPR5 Protein for Enhancing Plant Immunity
Source: Mol Plant Pathol. 2025 Feb 5;26(2):e70059. doi: 10.1111/mpp.70059 (PMC11798864; doi:10.1111/mpp.70059)
Supplement: Supplementary file 3 — FIGURE S3. The locations of the NAAIRS substitutions in the CPR5 protein sequence. [file MPP-26-e70059-s005.pdf]

1

ATGGAAGCCCTCCTCCTCCCTCCTTCGCCGGAACCCCAAAATCAAATCACCAATCCGGCGAATTCAAAGCCAAATCATCAATCTGGTGACGTACATAAAGATGAGACGATGATGATGAAG 120

M E A L L L P P S P E P Q N Q I T N P A N S K P N H Q S G D V H K D E T M M M K 40

2 3 4 5 6 7

AAGAAGAAGGATACGAATCCATCGAATTTGGAAAAGAGAAAACTCAAGGGAAAGAAGAAAGAGATTATGGACAACGACGAAGCTTCTTCGTCCTATTGTTCTACATCTTCTACCTCTAAT 240

K K K D T N P S N L E K R K L K G K K K E I M D N D E A S S S Y C S T S S T S N 80

8 9 10

TCAAATTCTACTAAAAGGGTTACGAGAGTGGTTCATAGATTACGAAACCCTATGCGGTTAGGTATGGCTCGACGAAGCGTTGGTGAACGACAAGCTGAAAAATTGGCGAAGCCTCTGGGC 360

S N S T K R V T R V V H R L R N P M R L G M A R R S V G E R Q A E K L A K P L G 120

11 12 13 14 15 16 17

TTTTCACTTGCCGCTTTTGCTAATATGGTTATTGCGAGAAAGAATGCCGCAGGTCAGAATGTTTATGTTGATGATCTTGTTGAGATCTTTGCTACTCTTGTCGAAGAATCATTAGCCAAT 480

F S L A A F A N M V I A R K N A A G Q N V Y V D D L V E I F A T L V E E S L A N 160

18 19 20 21 22

GTTTATGGTAATAAGCTTGGTTCCTTTGCGACCAACTTTGAGCAAACATTCAGCAGTACTCTAAAGATCCTTAAATTGACCAATGAATGTGCAAATCCACATCAGTCAAACAATAATGAT 600

V Y G N K L G S F A T N F E Q T F S S T L K I L K L T N E C A N P H Q S N N N D 200

23 24

GGTGGGAGTTGTAATTTAGATCGCTCTACCATAGACGGATGCTCAGACACCGAGCTATTTGAGAGGGAGACTTCATCTGCTACGTCTGCTTATGAAGTGATGCAAGGCAGTGCAACAGCA 720

G G S C N L D R S T I D G C S D T E L F E R E T S S A T S A Y E V M Q G S A T A 240

25 26 27 28 29 30 31

ACCTCTTTGATGAATGAGCTTGCCCTTTTCGAAGAGACTCTACAACTCTCTTGTGTCCCTCCTAGAAGTTCAGCAATGGCTTTGACCACAGACGAAAGGTTTTTAAAAGAGCAAACACGA 840

T S L M N E L A L F E E T L Q L S C V P P R S S A M A L T T D E R F L K E Q T R 280

32 33 34 35 36 37

GCAAACGACCTAAAGACCGTGGAGATTGGTCTTCAAATAAGAGAGTTAAGGTGCAAAGAGACGGCGCTAGGATTAAAATTTGAATCAAACAACCTGGGAAAGCGGCGCTAGAGTTGGAT 960

A N D L K T V E I G L Q I R E L R C K E T A L G L K F E S N N L G K A A L E L D 320

38 39 40

GTTTCGAAAGCTGCATTCAGAGCGGAGAAATTCAAAACCGAATTAGAAGATACAAGAAAAGAAGAGATGGTCACAAGAATCATGGATTGGCTCCTCGTAAGTGTCTTCAGCATGTTGGCT 1080

V S K A A F R A E K F K T E L E D T R K E E M V T R I M D W L L V S V F S M L A 360
