# Supplementary material for: Identification and Application of the Heptad Repeat Domain in the CPR5 Protein for Enhancing Plant Immunity
Source: Mol Plant Pathol. 2025 Feb 5;26(2):e70059. doi: 10.1111/mpp.70059 (PMC11798864; doi:10.1111/mpp.70059)
Supplement: Supplementary file 6 — FILE S1. Experimental procedures. [file MPP-26-e70059-s002.docx]

**EXPERIMENTAL PROCEDURES**

**Plant materials and growth conditions**

The *Arabidopsis thaliana* plants used in this study were in the Columbia (Col-0) background. The condition of growth chamber for Arabidopsis was set temperature at 22℃ and light intensity at 120 μmol m^–2^ s^–1^ generated by Philips Lifemax Cool White fluorescent bulbs with a 16-h light/8-h dark photoperiod. The *cpr5-1* (referred to as *cpr5*) mutant is as described (Bowling et al., 1997). The wild-type Upland cotton *Gossypium hirsutum* L. accession HM-1 was used in this study. The condition of growth room for cotton was set temperature at 25℃ under a 16-h light/8-h dark photoperiod.

**The CPR5 NAAIRS analysis (CNA)**

The CNA was carried out as described (Wilson et al., 1985). Briefly, the CNA constructs were generated by replacing the 18-bp nucleotide sequence of the *CPR5* gene with the sequence 5'-AATGCTGCTATACGATCG-3', which encodes six amino acids (NAAIRS). These constructs were subsequently introduced into *cpr5* mutants to evaluate the critical role of the six replaced amino acids in CPR5 function.

**Plant transformation**

The Agrobacterium strain used for plant transformation was GV3101. The transformation of Arabidopsis plants was performed using the floral dip method as described (Wang et al., 2014). Arabidopsis T0 seeds were sown in soil and, after one week, the herbicide Basta was applied every two days to screen for T1 transformants. The transformation of cotton plants was conducted by Towin Biotechnology (Wuhan, China). For the selection of cotton T0 transformants, kanamycin sulfate was used on agar plates. Subsequently, the company planted the T0 transformants and harvested the T1 seeds.

**Pathogen infection in Arabidopsis plants**

Infection on Arabidopsis plants with the virulent pathogen *Pseudomonas syringae pv. maculicola* ES4326 (*Psm*) and the avirulent pathogen *Psm* carrying the effector gene *AvrRpt2* (*Psm/AvrRpt2*), was carried out as described (Wang et al., 2014). Briefly, 4-week-old plants were used for pathogen infection. The bacteria were initially streaked onto King’s B medium supplemented with 100 mg/mL streptomycin and incubated at 30℃ for 2 days, with this process being repeated twice to rejuvenate the pathogen. To resuspend the bacteria, 10 mM MgSO₄ and four to six beads were added to the plate, followed by gentle agitation. The bacterial suspension was then diluted with 10 mM MgSO₄ to achieve a working concentration. A 1-mL syringe with a blunt-end needle was employed to inject the suspension into the abaxial surface of the leaves, with two leaves being injected per plant and 12 plants per genotype. Bacterial growth within the leaves was quantified at 3 days post-inoculation (dpi). Leaf disks (two per plant, from eight plants per genotype) were excised using a standard paper hole punch (8 mm diameter). These disks were placed in a 2-mL tube containing beads with 500 μL of 10 mM MgSO₄, and the samples were homogenized using a Geno-Grinder. Subsequently, 20 μL of the tissue suspension were transferred to a 96-well plate, which already contained 180 μL of 10 mM MgSO₄. Serial 10-fold dilutions were prepared by transferring 20 μL from the previous dilution into 180 μL of 10 mM MgSO₄. Aliquots of 10 μL from each dilution were streaked onto King’s B medium amended with 100 mg/mL streptomycin using an eight-channel pipette. The plates were incubated at 30℃ for 2 days, after which individual colonies from the most decipherable dilution were counted.

**Pathogen infection in cotton plants**

*Verticillium dahliae* strain V991 was cultured in potato dextrose broth (PDB) at a dilution ratio of 1:100 and incubated on a shaker at 25°C and 150 rpm. After 5 days, the spore suspension was filtered through gauze and then diluted with sterile water to achieve a concentration of 1×10^7^ spores/mL. Four-week-old Upland cotton plants, *Gossypium hirsutum* L. accession HM-1, with one pair of true leaves were inoculated with the suspension of *V. dahliae* spores, by applying the suspension to injured roots for two and a half minutes. Following inoculation, the plants were re-planted into soil and transferred to a growth chamber set at 25°C, under a 16-hour light/8-hour dark photoperiod and maintained at 80% humidity. The degree of plant wilt was assessed and categorized into five grades, from 0 to 4, according to the severity of disease symptoms at 12 dpi. Subsequently, the disease index was calculated as described (Yi et al., 2023).

**Reverse transcription-quantitative polymerase chain reaction (RT-qPCR)**

RNA was extracted using TRIzol Reagent (Invitrogen) and cDNA was synthesized using the TransScript One-Step gDNA Removal and cDNA Synthesis SuperMix (Cat. No. AH311-02, TransGen Biotech, Beijing, China). RT-qPCR was performed using the NovoStart SYBR qPCR SuperMix plus (Cat. No. E096, Novoprotein, Shanghai, China) in the CFX Connect Real-Time PCR System (BIO-RAD, Hercules, CA). *ACTIN 2* (*ACT2*, AT3G18780) was used as an internal control. Primers used for qPCR are listed in Table S1.

**Confocal laser scanning microscopy (CLSM)**

Images of the fluorescent proteins were obtained using a Zeiss LSM 5 PASCAL Confocal Laser Scanning Microscope (Carl Zeiss, Jena, Germany).

**Co-immunoprecipitation (Co-IP)**

Co-IP were conducted as described (Wang et al., 2014). Protein were immunoprecipitated (IPed) with anti-c-MYC magnetic beads (Cat. No. B26301, Selleck, Shanghai, China). Input and IPed proteins were detected with anti-MYC (Cat. No. R1208-1, HUABIO, Hangzhou, China), anti-FLAG antibody (Cat. No. AE005, ABclonal, Wuhan, China), and anti-HA antibody (Cat. No. AF0039, Beyotime, Shanghai, China).

**Statistical analysis**

Data were analyzed by one-way analysis of variance (ANOVA) with Bonferroni post hoc test or a two-tailed Student's t-test. The experiments were conducted in triplicate. The error bar in the graph represents the standard error of the mean (SEM). For the ANOVA results, the letter above the bar indicates a statistically significant difference between groups at P < 0.05. For the Student's t-test results, NS, not significant; *, P < 0.05; **, P < 0.01; ***, P < 0.001; ****, P < 0.0001.

**References**

Bowling, S. A., Clarke, J. D., Liu, Y., Klessig, D. F. & Dong, X. (1997) The cpr5 mutant of Arabidopsis expresses both NPR1-dependent and NPR1-independent resistance. *The Plant Cell*, 9, 1573-84.

Wang, S., Gu, Y., Zebell, S. G., Anderson, L. K., Wang, W., Mohan, R. & Dong, X. (2014) A noncanonical role for the CKI-RB-E2F cell-cycle signaling pathway in plant effector-triggered immunity. *Cell Host & Microbe*, 16, 787-94.

Wilson, I. A., Haft, D. H., Getzoff, E. D., Tainer, J. A., Lerner, R. A. & Brenner, S. (1985) Identical short peptide sequences in unrelated proteins can have different conformations: a testing ground for theories of immune recognition. *Proceedings of the National Academy of Sciences of the United States of America*, 82, 5255-9.

Yi, F., Song, A., Cheng, K., Liu, J., Wang, C., Shao, L., Wu, S., Wang, P., Zhu, J., Liang, Z., Chang, Y., Chu, Z., Cai, C., Zhang, X., Wang, P., Chen, A., Xu, J., Burritt, D. J., Herrera-Estrella, L., Tran, L. P., Li, W. & Cai, Y. (2023) Strigolactones positively regulate Verticillium wilt resistance in cotton via crosstalk with other hormones. *Plant Physiology*, 192, 945-966.
