# Supplementary material for: Identification and Application of the Heptad Repeat Domain in the CPR5 Protein for Enhancing Plant Immunity
Source: Mol Plant Pathol. 2025 Feb 5;26(2):e70059. doi: 10.1111/mpp.70059 (PMC11798864; doi:10.1111/mpp.70059)
Supplement: Supplementary file 7 — TABLE S1. The primers used for this study. [file MPP-26-e70059-s007.docx]

**TABLE S1** The primers used for this study.

| Primer name | Sequence (5’-3’) |
| --- | --- |

| ACT2-QPCR-F | GGCTCCTCTTAACCCAAAGGC |
| --- | --- |
| ACT2-QPCR-R | CACACCATCACCAGAATCCAG |

| PR1-QPCR-F | CTCATACACTCTGGTGGG |
| --- | --- |
| PR1-QPCR-R | TTGGCACATCCGAGTC |
| PR2-QPCR-F | CAGATTCCGGTACATCAACG |
| PR2-QPCR-R | AGTGGTGGTGTCAGTGGCTA |
